# Characteristics of the Soil Microbial Communities in Different Slope Positions along an Inverted Stone Slope in a Degraded Karst Tiankeng

**DOI:** 10.3390/biology10060474

**Published:** 2021-05-27

**Authors:** Cong Jiang, Jie Feng, Su-Feng Zhu, Wei Shui

**Affiliations:** 1 College of Urban and Environmental Sciences, Peking University, Beijing 100871, China; jcong@pku.edu.cn; 2 College of Environment and Resources, Fuzhou University, Fuzhou 350116, China; n180627027@fzu.edu.cn; 3 Chinese Research Academy of Environmental Sciences, Beijing 100020, China; zhu.sufeng@craes.org.cn

**Keywords:** refugia, high-throughput sequencing, functional diversity, spatial variation

## Abstract

**Simple Summary:**

Karst tiankeng is a special and magnificent surface negative terrain with unique scientific value. The underground forests developed on inverted stone slopes in tiankeng are important areas for biodiversity conservation. This research used Illumina high-throughput sequencing technology to determine the soil microbial communities at four sites (at the bottom of the slope (BS), in the middle of the slope (MS), in the upper part of the slope (US), and outside the tiankeng (OT)) along the inverted stone slopes. The microbial communities at different slope positions presented similar compositions but different abundances. The dominant phyla in the inverted stone slope were Proteobacteria, Actinobacteria and Acidobacteria. The microbial community diversity was greater at the US site. The microbial communities with more abundant functional genes involved in C/N cycles were located at the BS site. The distribution of the microbial community was highly correlated with the Total nitrogen and pH. Understanding the soil microbial communities on inverted stone slopes is important for monitoring the ecology of tiankeng and biodiversity value assessments.

**Abstract:**

The underground forests developed on inverted stone slopes in degraded karst tiankengs are important areas for biodiversity conservation, but the microbial community profiles have not been sufficiently characterized. Thus, we investigated the soil microbial communities at four sites (at the bottom of the slope (BS), in the middle of the slope (MS), in the upper part of the slope (US) and outside the tiankeng (OT)) in the Shenxiantang tiankeng. The dominant phyla in the inverted stone slope were Proteobacteria, Actinobacteria, and Acidobacteria, and the relative abundance were different in different slope positions. The Shannon–Wiener diversity index of the microbial community was significantly greater for the US site than for the MS or BS sites. The metabolism functional pathways (including C/N cycle) were more abundant at the BS site. Total nitrogen and pH were the dominant factors in determining the distribution of the microbial community along an inverted stone slope. These results suggest that topographic heterogeneity can influence the variations in the soil microbial structure, diversity, and function in degraded karst tiankengs and emphasized the ecological value of inverted stone slopes within karst tiankengs.

## 1. Introduction

The karst tiankeng was named by Zhu in 2001 and was defined as a kind of large open pit more than 100 m in width and depth, with a large volume and steep rock walls, and its bottom connected with an underground river [1]. Due to their vertical rock wall linings, the habitats inside and outside tiankengs are relatively independent [2,3]. The habitats inside tiankengs have unique microclimates and high-quality hydrothermal conditions and soil properties. Many studies have shown that tiankengs act as refugia for biodiversity amid changing global climates [4,5]. Currently, more than 200 karst tiankengs have been discovered globally, with more than 70% distributed in China. According to the evolution of the geological environment, karst tiankengs can be divided into primary tiankengs, mature tiankengs, and degraded tiankengs [6]. Degraded tiankengs are the most widely distributed among the different tiankeng types and are characterized by incomplete margins, high accessibility, and close connections with outside areas.

Soil microbes participate in biogeochemical processes and the soil nutrient cycle. Microbial community structure and diversity can reflect changes in soil ecological processes and have an impact on surface vegetation [7]. To date, the heterogeneity of the soil microbial community has been studied in desert [8], forest [9] and grassland ecosystems [10], but few studies have addressed these communities in hillslope ecosystems. In particular, the underground hillslopes in karst tiankengs have not been investigated. On sloping lands, soil substrates and soil water may exhibit obvious changes even over short distances [11,12]. The varying soil conditions along a slope will further affect the distinctive vegetation coverage and microbial communities at different topographic positions [13]. An inverted stone slope is an accumulation of blocks formed by the collapse of tiankeng cliffs. In a degraded tiankeng ecosystem, inverted stone slopes play an important ecological role. In addition, inverted stone slopes serve as bridges, connecting the inside and outside of the tiankeng, and the soil conditions and microclimates at different slope positions have obvious differences. Understanding the soil microbial communities on inverted stone slopes is important for monitoring the ecology of tiankengs and biodiversity value assessments.

Here, we used Illumina high-throughput sequencing technology to determine the spatial variations in the microbial species along the slope studied and to analyze the similarities in the microbial community composition among the different slope positions. Alignment with the KEGG database was used to predict the potential functional types of the microbial communities. The objectives were to (1) characterize the variation in the soil microbial community diversity, structure, and function along an inverted stone slope in a degraded karst tiankeng, and (2) identify the environmental variables that are the main factors in determining the microbial communities.

## 2. Materials and Methods

### 2.1. Study Area

**Figure A1:**
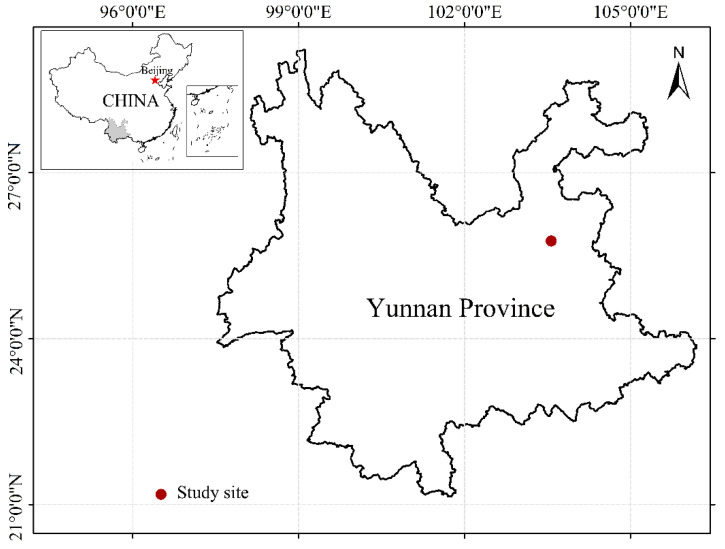
Location of the study site in the Shenxiantang tiankeng in Yunnan Province, China.

The Shenxiantang tiankeng (25°48′11.2′′ N, 103°34′45.8′′ E) is located in the Haifeng Nature Reserve, Yunnan Province, in the northeast of the Zhanyi Tiankeng Group (Figure A1). The rocks are dominated by carbonate and dolomite, and the soil belongs to the red soil horizon of Yunnan soils. The climate in the region is a typical subtropical plateau monsoon climate type with an average annual temperature of 13.8–14 °C, annual rainfall of 1073.5–1089.7 mm, annual evaporation of 2069.1 mm, relative humidity of 71%. The regional climate is characterized by distinct dry and wet seasons, i.e., dry and windy in winter and spring, and hot and rainy in summer and autumn. The Shenxiantang tiankeng is found at an elevation of 2028–2031 m; it has a long diameter of 421.9 m and a short diameter of 348.7 m, with a depth of 148.7 m. According to the tiankeng degradation rating index (Table A1 in Appendix A), the Shenxiantang tiankeng was classified as moderately degraded. The bottom of the Shenxiantang tiankeng has been in a natural state for more than ten years, after being restored from cultivated land (corn and tobacco). The north and south sidewalls of this tiankeng are completely degraded and formed from inverted stone slopes. The vegetation on the north side is dominated by sparse shrubs, whereas the vegetation on the south side is more abundant, dominated by shrubs and arbors vegetation. The west side is an undegraded vertical exposed wall, and the east side is a partially degraded tiankeng wall.

### 2.2. Sample Collection

On the south side of the Shenxiantang tiankeng, along the inverted stone slope, a sampling transect was established from the bottom of the slope to the top of the slope. The sampling sites (the bottom of the slope (BS), the middle of the slope (MS), the top of the slope (US), and outside the tiankeng (OT)) were selected for comparison. Each sampling site consisted of a 20 × 20 m (m^2^) plot. The plant coverage, species were recorded to calculate the Shannon-Wiener index. Three 5 × 5 m (m^2^) small squares were set along the diagonal in the large square to collect soil samples. Soil samples were collected by the five-point method: large pieces of debris were removed, and surface soil samples from a depth of 0−10 cm were collected. The five soil samples were merged to form a single sample. The soil samples were sieved with a 2 mm sieve and stored in a cooler. After returning to the laboratory, the soil samples were divided into two parts. One part was used to determine the physical properties of the soil, and the other part was preserved at −80 °C and used for environmental DNA extraction and microbial diversity analysis. The soil water content (SWC), soil organic carbon (TOC), total nitrogen (TN), total phosphorus (TP), total potassium (TK), and soil pH values were measured as previously described [14].

### 2.3. DNA Extraction and Sequancing

We used the E.Z.N.A.^®^ Soil DNA Kit (OMEGA, Norcross, GA, USA) to extract microbial DNA from soil following the manufacturer’s directions. The integrity and concentration of the DNA were assessed by 1% agarose gel electrophoresis and fluorometer. An initial total amount of 500 ng DNA was fragmented to an insert length of approximately 500 bp using Covaris M220 (Sangon, Shanghai, China) for paired-end library construction. The paired-end library was constructed according to the instructions of the NEB Next^®^ UltraTM DNA Library Prep Kit (Illumina, San Diego, CA, USA). Paired-end sequencing was performed by the Illumina HiSeq 4000 platform (Illumina, San Diego, CA, USA) at Sangon Biotech Co., Ltd. (Shanghai, China) using a HiSeq 3000/4000 PE Cluster Kit and HiSeq 3000/4000 SBS Kits according to the manufacturer’s instructions (www.illumina.com, accessed on 25 October 2019). Library size and length distribution were detected by 2% agarose gel electrophoresis and an Agilent Technologies 2100 DNA 1000 Kit. The library concentration determination was performed using a Thermo Qubit 4.0 fluorometer (Q33226, Thermo Fisher, Waltham, MA, USA).

### 2.4. Processing of Sequencing Data

The original image data file obtained by Illumina Hiseq™ is analyzed by Base Calling and converted into the raw Data. Perform statistics on raw data quality values and use FastQC (version 0.11.2) to visually evaluate the quality of the sequencing data of the samples. Trimmomatic was used to remove low-quality reads and get clean reads (having N bases or with a quality value <20 or connector sequence in reads or length < 50 bp) [15]. The stitching software IDBA_UD based on the De Bruijn graph principle was used to obtain contigs [16]. Each contig of the open reading frames (ORFs) prediction was created using Prodigal, and ORFs (length ≥ 100 bp) were chosen and translated to amino acid sequences. Clustering 95% sequence identity (90% coverage) of the whole predicted gene sequences catalog was performed using CD-HIT (version 4.6), and the longest genes of every cluster were selected to construct a nonredundant gene catalog. Use Bowtie2 (version 2.1.0) to compare the clean reads of each sample with the nonredundant gene catalog, use Samtools (version 0.1.18) to count the number of reads on each gene being compared, and calculate the abundance of each gene in the sample according to the length of the gene, and summarize the gene abundance of all samples [17]. The microbial community taxonomic annotation was performed using BLASTP (BLAST version 2.2.21, http://blast.ncbi.nlm.nih.gov/Blast.cgi, accessed on 2 November 2019) (e-value ≤ 1 × 10^−5^, score > 60). Gene species classification annotation information was obtained according to NCBI’s microbial taxonomy information database (http://ncbi.nlm.nih.gov/, accessed on 2 November 2019). The hypothetical amino acid sequences were compared against the KEGG pathway database (http://www.kegg.jp, accessed on 2 November 2019) using GhostKOALA (version 1.0), and KO numbers and pathways annotation information was obtained. The sequence results were submitted to the SRA at NCBI under the accession number PRJNA640943.

### 2.5. Statistical Analysis

The alpha indices (Shannon–Wiener) of the soil microbial community were determined with QIIME (version 1.9.0) [18]. Linear discriminant analysis effect size (LEfSe) was used to detect the microbial biomarkers (http://huttenhower.sph.harvard.edu/galaxy/root?tool_id=PICRUSt_normalize, accessed on 3 November 2019). Principal coordinates analysis (PCoA) was used to analyze the microbial community composition similarities based on a Bray-Curtis matrix calculated in QIIME (version 1.9.0) [18]. The analysis of similarities (ANOSIM) was performed by the R vegan package [19]. The soil physiochemical properties influencing the microbial communities were generated by multivariate redundancy analysis (RDA), which was performed in Canoco (version 5 for windows; Ithaca, NY, USA). The Mantel test was performed in QIIME (version 1.9.1) [18]. Heatmaps and Venn diagrams were created using custom R scripts.

## 3. Results

### 3.1. Characteristics of the Environmental Factors among Slope Positions

The characteristics of the environmental factors, including the plant diversity and soil characteristics, are summarized in Table 1, Table A2 and Table A3. The Shannon–Wiener index of the plants was significantly lower OT site than inside it and was highest at the US site, with no significant difference between the US and MS sites (*p* > 0.05). BS sites were mainly distributed with herbs and a few shrubs; MS mainly distributed wit shrubs dominated by *Myrsine africana* Linn and *Debregeasia orientalis* Chen, C.J.; and US were mainly distributed with arbor dominated by *Cyclobalanopsis glauca* (Thunb) Oerst and *Keteleeria evelyniana* Mast. The OT sites were mainly distributed with shrub dominated by *Myrsine africana* Linn and *Viburnum propinquum* Regarding the soil samples from the different slope sites, the SWC was significantly higher at the US site (*p* < 0.05). The SOC and TN varied little among slope positions inside the tiankeng sites but were significantly lower in OT site. The TP at the BS site was 0.64 g/kg greater than that at the MS and US sites, with no significant difference between the MS and US sites. The TK was higher at the BS site than other sites. The soil pH ranged from 6.30 to 6.79, with no significant difference between the BS and MS sites.

### 3.2. Composition and Diversity of the Microbial Community along the Slope

#### 3.2.1. Composition of the Microbial Community

In total, 675 million total sequences were obtained (43 million to 104 million per sample). The raw reads of the samples ranged from 6.5 to 15.6 Gb. The clean reads after data quality control accounted for about 90% of the raw reads. A total of 3.3 million contigs and 4.9 million ORFs were obtained from 12 samples.

**Figure 1:**
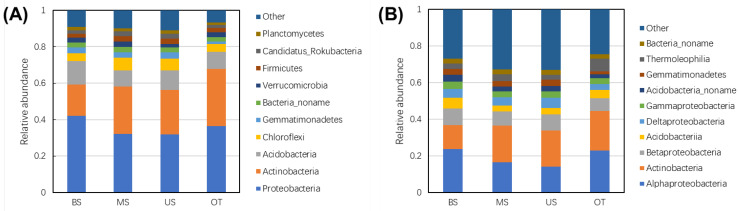
Microbial community composition of different sample site. (**A**) at the phyla level, (**B**) at the classes level.

**Figure A2:**
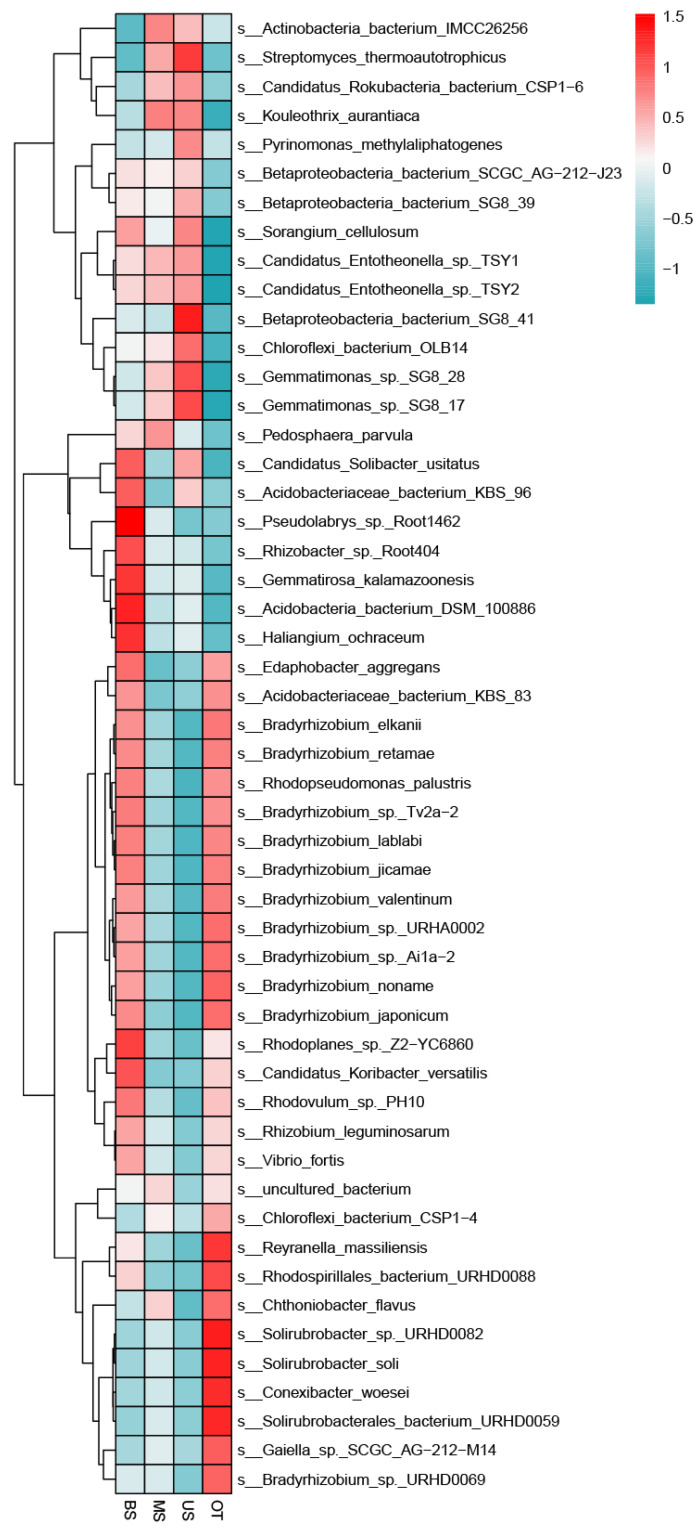
Microbial community composition of different sample site at the species level.

According to metagenomic sequence analysis, a total of 6 kingdoms, 106 phyla, 195 classes, 371 orders, 709 families, 2326 genera, and 13,862 species were obtained. At the domain level (Table A4), the highest relative abundance of the tiankeng microbial community was bacteria, followed by archaea, fungi and viruses. The microbial composition at the phylum and class level (top 10) is shown in Figure 1A. At the phylum level, the microbial communities at different sites presented similar compositions but different abundances. Four phyla (Proteobacteria, Actinobacteria, Acidobacteria, and Chloroflexi) together accounted for more than 70% of the relative abundance in each site samples. Proteobacteria was the most abundant phylum at all of the sites with the relative abundance of 41.99% (BS), 32.09% (MS), 31.87% (US), and 36.40% (OT). The second most abundant phylum was Actinobacteria, and the relative abundance was 17.19%, 25.96%, 24.42%, and 31.41% in BS, MS, US, and OT sites, respectively. The other dominant phyla were Acidobacteria (9.05%−13.00%) and Chloroflexi (4.19%−6.89%). Except for the top 10 abundant phyla, the relative abundance of “other phyla” were 9.20% (BS), 10.01% (MS), 11.02% (US), and 6.81% (OT). In “other phyla”, 11 archaea, 10 fungi, and 1 virus phyla were annotated (Table A5). The most abundant archaea phyla were Euryarchaeota, Candidatus_Bathyarchaeota and Crenarchaeota. The fungi phyla included Basidiomycota, Ascomycota, and Chytridiomycota. At the class level (Figure 1B), the bacterial communities were mainly assigned to Alphaproteobacteria, Actinobacteria, Betaproteobacteria, Acidobacteriia, and Deltaproteobacteria. Alphaproteobacteria was the most abundant class and accounted for a high proportion (14.01%–23.75%) of the microbial community, followed by Actinobacteria (12.96%–21.63%). The Actinobacteria and Betaproteobacteria varied at different sites. For example, Actinobacteria was more abundant in the OT site (21.63%). *Betaproteobacteria* was more abundant in the BS site (9.20%). In addition, Gammaproteobacteria, Acidobacteriia and Thermoleophilia were the main classes at different tiankeng sites. A heat map of the top 50 species is shown in Figure A2. The distribution characteristics of detected species were different along the tiankeng slope position. The most abundant species were *Acidobacteria***_***bacterium_DSM_100886* (1.59%−3.00%), *Rhodoplanes_*sp.*_Z2-YC6860* (1.34%−2.49%), and *Candidatus_Rokubacteria_bacterium_CSP1−6* (1.69%−2.77%). Nine of the top 50 species, including *Reyranella_massiliensis*, *Solirubrobacter_*sp.*_URHD0082*, *Solirubrobacter_soli,* and *Solirubrobacterales_bacterium_URHD0059* were more enriched in OT sites.

**Figure 2:**
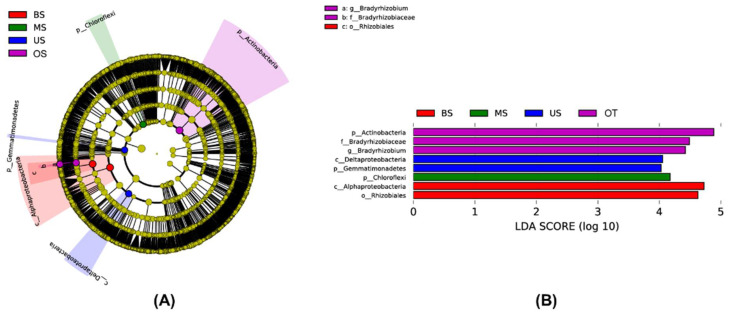
The LEfse analysis of microbial community of different sample site (**A**). The histogram of LDA scores with a threshold value of 4.0 (**B**).

The LEfSe analysis showed that the seven microbial clades exhibited significant differences along the tiankeng slope position (LDA score >4.0) (Figure 2). There were two, one, one, and three microbial clades in BS, MS, US, and OT, respectively (Figure 2B). Most differential microbial clades were enriched in OT. In particular, Alphaproteobacteria (class) and Rhizobiales (order) were specific to the BS; Chloroflexi (phylum) was specific to MS; Gemmatimonadetes (phylum) was specific to US; Actinobacteria (phylum), Bradyrhizobiaceae (family), and *Bradyrhizobium* (genus) were specific to OT.

#### 3.2.2. Diversity of the Microbial Community

**Figure 3:**
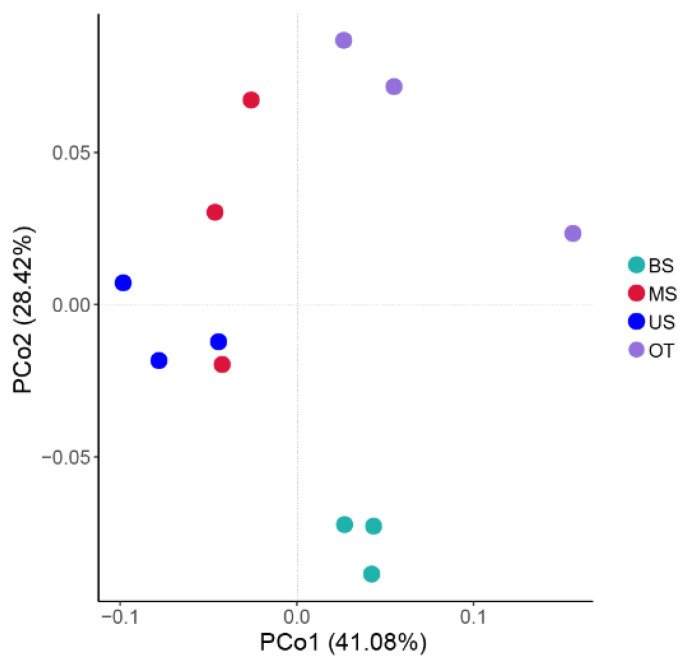
The principal coordinate analysis (PCoA) of microbial community for different sample site.

**Figure A3:**
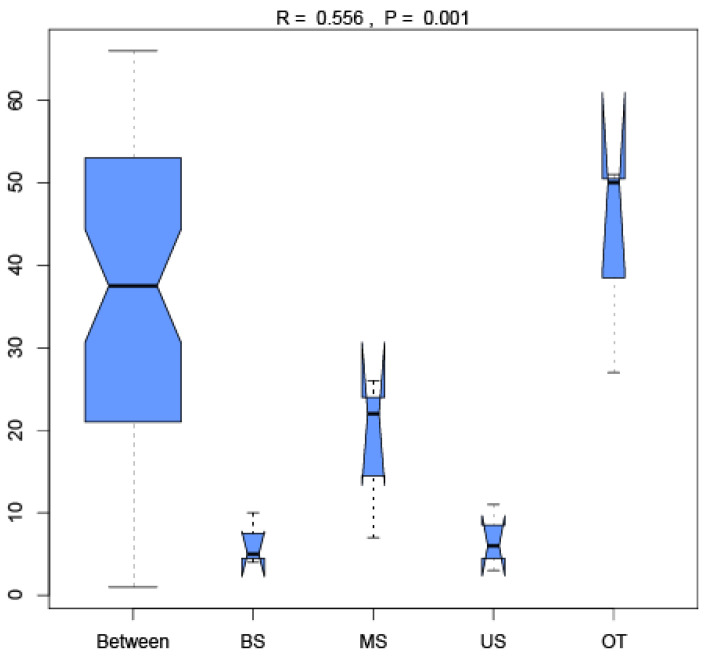
The analysis of similarities (AMOSIM) of microbial community for different sample sites.

The slope position affected the Shannon–Wiener index of the microbial community, and the highest Shannon-Wiener diversity was observed at the US site. In general, the Shannon-Wiener diversity indexes followed the trend of: US > MS > BS > OT (Table A6). The ANOSIM results showed that the microbial communities differed significantly (*r* = 0.556, *p* = 0.001) among the different slope positions (Figure A3). The PCoA results (Figure 3) showed that there were comparatively high discrepancies among the microbial communities between the different tiankeng slope positions, with the MS and US sites clustered closely and separately from the BS and OT sites; PCo1 and PCo2 explained 41.08% and 28.42% of the variation.

### 3.3. Functional Differences in Microbial Communities along the Slope

**Figure 4:**
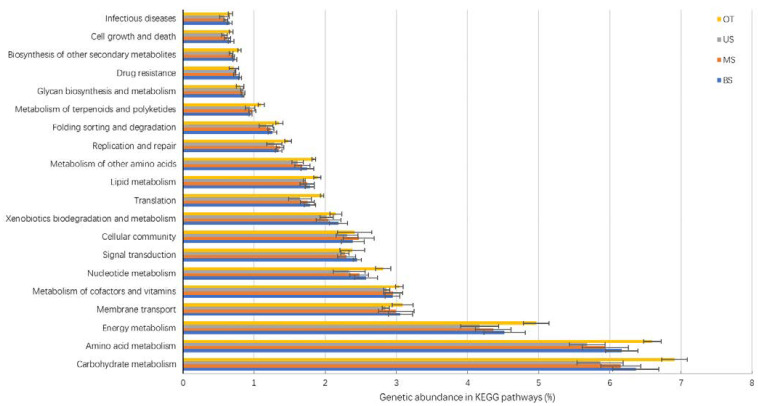
The abundance of genes associated with KEGG pathways of different sample sites.

**Figure 5:**
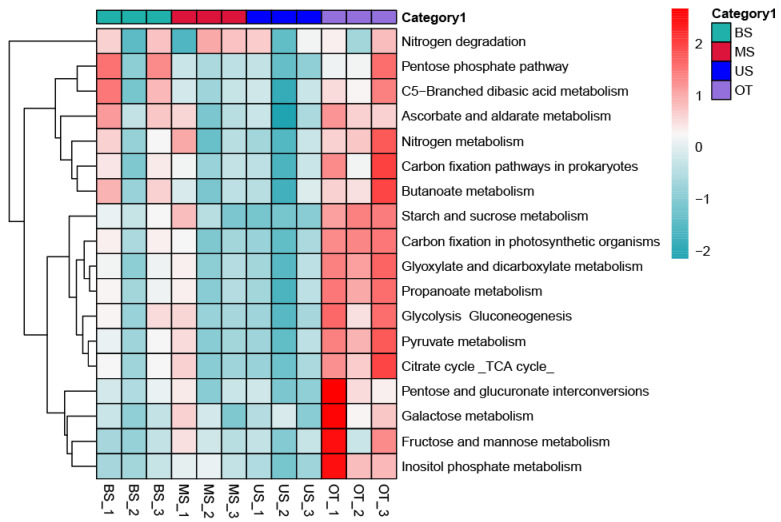
The abundance of genes associated with C and N cycle of different sample sites.

**Figure A4:**
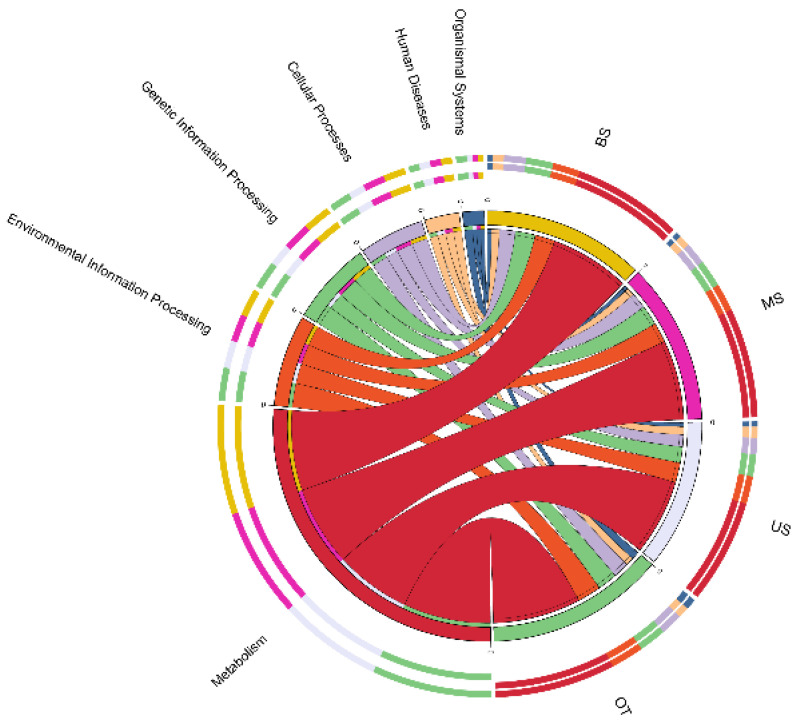
Microbial community function pathways of different sample sites.

To explore the functional differences in the microbial communities along the tiankeng slope, the metagenome was annotated with the metabolic cycles and pathways in the KEGG database (Figure 4 and Figure 5 and Figure A4, Table A7). The six functional categories included metabolism (range: 53.72%−54.06%), environmental information processing (range: 14.07%−14.59%), genetic information processing (range: 12.6%−13.0%), cellular processes (range: 9.45%−9.67%), human diseases (range: 5.38%−5.58%), and organismal systems (range: 3.76%−3.85%) (Figure A4). A total of 3877 KO genes were annotated across all of the soil samples, and the 20 most abundant functional pathways are listed in Figure 4. Among the top 20 pathways, the OT site had the highest genetic abundance in 15 pathways (e.g., carbohydrate metabolism, translation, and infection diseases). On the slope gradient, the genetic abundance of 15 pathways showed the trend of BS > MS > US. Based on the KEGG database (carbon and nitrogen cycle), microorganisms are more involved in the carbon fixation pathways in prokaryotes, pyruvate metabolism, and glyoxylate and dicarboxylate metabolism pathways related to the C cycle. The genes related to the C and N cycles exhibited the highest abundances in OT sites and decreased in gene abundances as the slope position rose (Figure 5).

### 3.4. Relationship between the Microbial Community and Soil Characteristics

**Figure 6:**
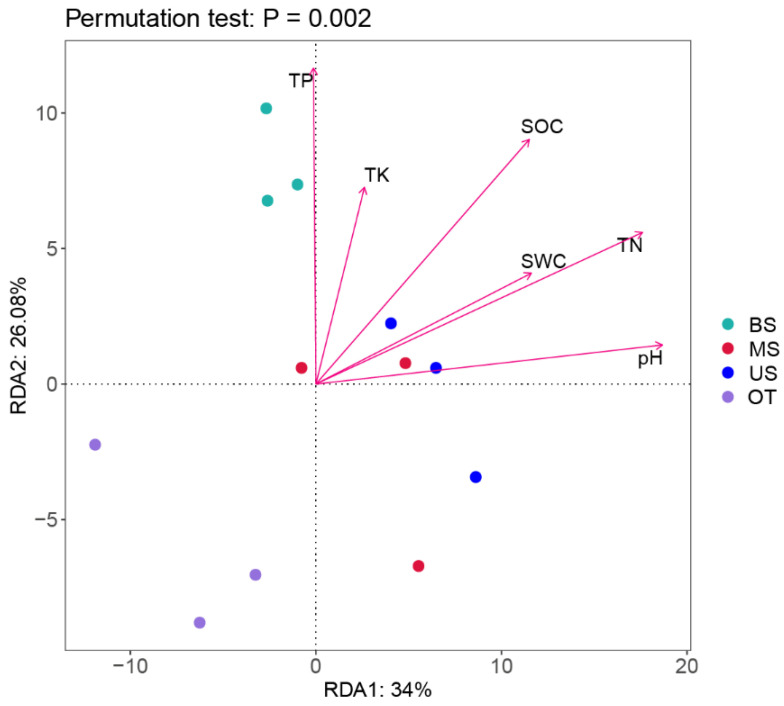
The redundancy analysis (RDA) of the soil microbial community with soil characteristics of different sample sites. Soil water content, SWC; Total organic carbon, TOC; Total nitrogen, TN; Total phosphorus, TP; Total potassium, TK.

The relationship between the microbial community and soil characteristics was analyzed using redundancy analysis (RDA). The RDA axes explained 60.08% of the total variations in the microbial community (Figure 6). pH, TN and SOC were the key soil characteristics and were significantly correlated with the microbial communities (*p* < 0.05). Furthermore, the results of a Mantel test were used to discern the correlations among the UniFrac distances of the microbial community and the Bray–Curtis distance matrix of soil characteristics, and TN and pH were significantly related to differences in the microbial community along the different slope positions (*p* < 0.01) (Table A8).

**Figure A5:**
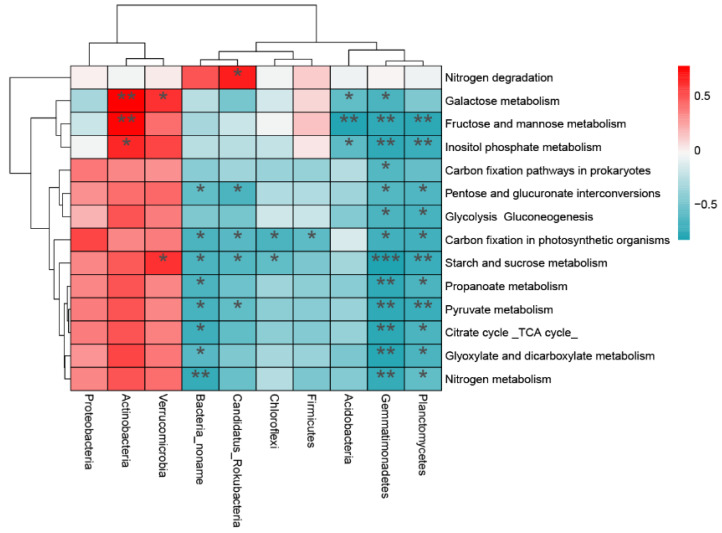
The correlation between the relative abundance of C/N cycle and top 10 microbial phyla. * correlation significant at the 0.05 level, ** correlation significant at the 0.01 level, *** correlation significant at the 0.001 level.

The change in C/N metabolic pathway gene abundance along the slope position gradient is significantly related to the abundance of microbial communities (Figure A5). Gemmatimonadetes and Planctomycetes were significantly related to most C/N metabolic pathway genes. Bacteria_noname, Candidatus_Rokubacteria, and Chloroflexi were significantly negatively correlated with carbon fixation in photosynthetic organisms and starch and sucrose metabolism. However, Actinobacteria was significantly positively with galactose metabolism, fructose and mannose metabolism, and inositol phosphate metabolism.

## 4. Discussion

### 4.1. Diversity and Composition of Microbial Communities along a Slope Gradient

Change in biodiversity with topography has been an important issue in biodiversity research in recent years [20]. Topography is among the decisive factors affecting the distribution pattern of species diversity [21]. As an important topographical factor, slope position can affect microbial community structure and diversity through changes in microclimate, soil properties, and vegetation characteristics [13]. Pu et al. found that in the tiankeng ecosystem, slope is a key factor affecting the composition of the microbial community [4]. The Shenxiantang tiankeng underground inverted stone slope provides a unique area for the investigation of the diversity and composition of microbial communities and related factors. In our study, slope position altered the composition and diversity of the microbial community. This result is consistent with that of research that reported that the bacterial communities significantly differ on slope gradients in Guangxi and Shenmu tiankengs and on the Loess Plateau [4,20]. The Shannon–Wiener index was greater in the US site than in the BS or MS sites, which clearly demonstrated the sensitivity of the microbial community diversity to spatial variation along the slope gradient. Previous studies have reported that soil erosion can cause soil deposition in bottom slope positions and drive substrate redistribution along the slope, leading to an obvious pothole effect [22]. However, our research found that the soil properties at the US site were more beneficial for microbiota than those at other sites. Karst landforms are rich in carbonate rocks and prone to dissolution. The field investigation found that the underground inverted stone slope experienced a secondary collapse, leading to the formation of a platform at the US site. The gentler slope allows the US site to receive more nutrients and thus creates more suitable habitats. Moreover, the US site has higher plant diversity than the other sites (Table A2). Higher levels of plant diversity are associated with more litter and root exudates, which support enriched microorganism communities [23]. Previous studies also have shown that vegetation type is the driving factor that affects the metabolic activity, structure, and composition of soil microbial communities [24]. With the change in slope position (BS to US), vegetation gradually succeeded from mainly herbs to mainly arbors. As the change of surface vegetation, the composition and structure of soil microbial communities also change. The Shannon–Wiener index of the microbial community increased with succession until the arbor stage (US), which could be partially due to the increase in soil nutrient (Table 1).

The microbial communities on the inverted stone slope in Shenxiantang tiankeng were dominated by Proteobacteria and Actinobacteria. Our results are consistent with previous research conducted in Shengmu tiankeng [4]. Proteobacteria play important roles in ecological value and are involved in energy metabolism [25]. The other most abundant phyla included Acidobacteria and Chloroflexi. Acidobacteria and Chloroflexi are considered to play a key role in nutrient cycles [26]. This result indicates that microorganisms with strong organic degradation and metabolic activity may survive well in the tiankeng ecosystem. The differences in the abundance of these phyla may indicate that Proteobacteria, Actinobacteria, and Acidobacteria were affected by habitat conditions such as soil properties and vegetation changes. At the class level, Alphaproteobacteria was the most abundant and higher in BS. The following dominant classes were Actinobacteria and Betaproteobacteria, which were higher in OT and BS, respectively. At the species level, the dominant species were similar in composition but different in relative abundance. These results may reflect the sensitivity of microbial community composition and diversity to spatial changes along the inverted stone slope in Shenxiantang tiankeng. In addition, the LDA results further confirmed that the samples from the different slope position sites and the sites outside the tiankeng had their own biomarkers, which was also supported by PCoA results.

### 4.2. Comparisons of Microbial Community Functional Groups among the Slope Gradient

The microbial genes were compared with the KEGG database to determine the functional groups of the microbes that occurred among the slope gradients. The gene families within the microbial community differed with slope position. Changes in microbial community composition are often associated with variations in microbial community function [27]. In our study, the microbial community was involved in diverse functional pathways. Among the slope gradients, the relative abundance of most functional pathways showed the trend of BS > MS > US (Figure 4). Similar to the functional pathways, the genes related to the C/N cycles were higher in BS and decreased as the slope position rose. In view of the basic role of the microbial community in the ecosystem, changes in surface vegetation also lead to changes in the function of the microbial community [28]. Higher plant diversity can increase the soil nutrient input and turnover rate of plant biomass, and therefore change the microbial communities functions in nutrient cycling processes [29]. Moreover, given that the plant litter is the main source of soil organic carbon in the ecosystem, vegetation type may also influence microbial community functions [24]. The arbors in US sites can provide more C sources for microbes. Field investigations also found that the surface layer of soil at the US sites had accumulated more litter. Compared with US sites, the microbe growth of BS sites is more likely to be restricted by soil nutrition. The high abundance of metabolism pathways could help complete the efficient decomposition of organic matter and conversion of soil nutrients [30]. However, the soil nutrients in the MS and US were relatively sufficient (this fact is supported by the soil characteristics shown in Table 1); microbes can easily obtain soil nutrients and cause the decline in the relative abundance of metabolism pathways. Overall, the abundance of metabolic functions (including C/N cycle) of microbes showed a downward trend from BS to US.

### 4.3. Relationships between Soil Characteristics and Microbial Communities

Previous research has proven that soil characteristics are key factors shaping soil microbial communities [31]. PCoA results showed the differences in microbial communities in different slope positions. This finding is consistent with previous research on the Shenmu tiankeng, in which it was founded that the composition and structure of the microbial community in the tiankeng were affected by the micro-habitat and showed obvious differentiation characteristics [4]. The RDA results showed that pH, TN, and SOC were the critical factors driving the microbial community structure. Moreover, Mantel test results showed that the pH and TN were the critical drivers of microbial community structure. In general, the soil microbial communities on the inverted stone slope at the tiankeng were mainly restricted by pH and TN. pH is a critical factor affecting soil microbial communities in different types of soil [32,33]. Pu et al. showed that microbial community structure was influenced by pH in the Shenmu tiankeng [4]. Soil TN is an important factor affecting microbial communities, so soil TN availability often limits microbial growth [34]. Due to differences in TN at the different slope positions, TN contents is the limiting factor of microbial communities. In addition, there was a significant difference in TN inside and outside the tiankeng, which may have affected the microbial communities.

The ecosystem function of soil microorganisms is inseparable from the characteristics of microbial community composition. The relative abundance of Acidobacteria, Actinomycetes, Gemmatimonadetes and Planctomycetes in the soil microbial community had a significant correlation with the abundance of C/N metabolic pathway, indicating that these microorganisms have a significant impact on the microbial community function. Previous studies have also confirmed that these microorganisms play an important role in the processes of the elemental biological earth cycle, plant residue degradation, and organic matter turnover [35].

## 5. Conclusions

In this karst tiankeng ecosystem, the microbial community composition, diversity, and function had divergent responses to spatial variations. The microbial communities at different slope positions presented similar compositions but different abundances. The dominant phyla in the inverted stone slope were Proteobacteria, Actinobacteria, and Acidobacteria. The microbial community diversity was greater at the US site. Based on the KEGG database, the microbial communities were mainly involved in carbohydrate metabolism and amino acid metabolism, and the microbial communities with more abundant functional genes involved in C/N cycles were located at the BS site. The distribution of the microbial community was highly correlated with the pH and TN. Our results showed the distribution characteristics and potential functional profiles of microorganisms on an inverted stone slope in a karst tiankeng and improved our understanding of the vertical differentiation of microorganisms. As a bridge connecting the inside and outside of tiankengs, inverted stone slopes form a unique open niche on which underground forests are found. The change in soil conditions with slope position can promote differences in the diversity, structure, and function of small-scale microbial communities. The analysis of microbial communities allowed us to better understand the various interactions that occur at the system level and offers advantages for ecological restoration and species protection in ecologically fragile karst areas.

## Figures and Tables

**Table 1:** Soil characteristics at different sampling sites.

Characteristics	BS	MS	US	OT
Soil water content (%)	43.21% ± 0.03 ab	42.45% ± 0.02 ab	48.46% ± 0.02 a	39.47% ± 0.02 b
Total organic carbon (g/kg)	44.43 ± 3.05 ab	42.67 ± 2.94 ab	48.90 ± 4.76 a	35.50 ± 3.25 b
Total nitrogen (g/kg)	2.69 ± 0.14 ab	2.77 ± 0.34 a	2.73 ± 0.11 a	2.13 ± 0.23 b
Total phosphorus (g/kg)	0.64 ± 0.09 a	0.43 ± 0.17 a	0.54 ± 0.11 a	0.39 ± 0.07 a
Total potassium (g/kg)	17.53 ± 0.93 a	15.97 ± 3.02 a	14.37 ± 4.43 a	13.51 ± 2.08 a
pH	6.57 ± 0.15 ab	6.72 ± 0.21 ab	6.79 ± 0.14 a	6.30 ± 0.15 b

Note: Different lowercase letters indicate significant differences at the 0.05 level.

## Data Availability

The sequence results were submitted to the SRA at NCBI under the accession number PRJNA640943.

## Appendix A

**Table A1:** Degradation classification of karst tiankengs.

Item	Light Degradation	Moderate Degradation	Severe Degradation	Heavy Degradation
Depth-width rate	(0.45, 1)	(0.35, 0.45)	(0.1, 0.35)	(0, 0.1)
Damage degree of wall area	0–20%	21%–50%	51%–80%	>81%
Damage degree of wall area	<1	1~2	3	>4(Circularity distribution)
Trapping	Good trapping	General trapping	Slightly poor trapping	Poor trapping
Pattern of pithead	Approximately ellipse	Irregular ellipse	Irregular polygon	Approximately large doline

**Table A2:** Sample locations and coordinates.

Sampl Sites	Geographic Coordinate		Altitude (m)	Plant Shannon–Wiener
	Longitude (E)	Latitude (N)		
BS	103°34′46.67”	25°48′7.75”	1949.2	1.76 ± 0.24 ab
MS	103°34′46.86”	25°48′6.90”	1972.9	2.04 ± 0.36 a
US	103°34′46.88”	25°48′7.34”	1998.3	2.19 ± 0.18 a
OT	103°34′45.93”	25°48′3.63”	2052.6	1.65 ± 0.08 b

Note: Different minuscule alphabet indicate divergence is significantly different at 0.05 levels.

**Table A3:** Plant characteristics of different sample sites.

Sample Sites	Plant	
	Dominant Species	Number of Species
BS	*Myrsine africana* Linn. (Shrub)	145
MS	*Myrsine africana* Linn. (Shrub)	216
	*Debregeasia orientalis* C. J. Chen (Shrub)	25
	*Ternstroemia gymnanthera* (Wight et Arn.) Beddome (Shrub)	18
	*Swida oblonga* (Arbor)	19
US	*Cyclobalanopsis glauca* (Arbor)	41
	*Keteleeria evelyniana* Mast. (Arbor)	40
	*Fraxinus griffithii* C. B. Clarke (Arbor)	10
OT	*Myrsine africana* Linn. (Shrub)	132
	*Viburnum propinquum* (Shrub)	28

**Table A4:** The relative abundance of microbial community at the domain level of different sample site.

	BS	MS	US	OT
Archaea	0.68%	0.82%	0.78%	0.50%
Bacteria	98.91%	98.94%	98.90%	99.29%
Fungi	0.37%	0.21%	0.30%	0.17%
Viruses	0.03%	0.03%	0.02%	0.03%

**Table A5:** The relative abundance of archaea, fungi, and viruses at the phyla level of different sample sites.

		BS	MS	US	OT
Archaea	*Euryarchaeota*	0.3851%	0.4911%	0.5203%	0.3353%
	*Candidatus_Bathyarchaeota*	0.0439%	0.0579%	0.0612%	0.0348%
	*Archaea_noname*	0.0406%	0.0426%	0.0469%	0.0250%
	*Crenarchaeota*	0.0262%	0.0376%	0.0377%	0.0235%
	*Candidatus_Thorarchaeota*	0.0083%	0.0107%	0.0107%	0.0068%
	*Candidatus_Lokiarchaeota*	0.0043%	0.0045%	0.0059%	0.0043%
	*Candidatus_Korarchaeota*	0.0013%	0.0015%	0.0013%	0.0014%
	*Candidatus_Micrarchaeota*	0.0003%	0.0001%	0.0003%	0.0002%
	*Candidatus_Nanohaloarchaeota*	0.0003%	0.0004%	0.0004%	0.0004%
	*Candidatus_Parvarchaeota*	0.0001%	0.0002%	0.0001%	0.0003%
	*Nanoarchaeota*	0.0000%	0.0002%	0.0000%	0.0001%
Fungi	*Basidiomycota*	0.2812%	0.0850%	0.0691%	0.0969%
	*Ascomycota*	0.0992%	0.1978%	0.1201%	0.1283%
	*Thaumarchaeota*	0.0845%	0.1292%	0.1366%	0.0657%
	*Fungi_noname*	0.0090%	0.0087%	0.0095%	0.0065%
	*Chytridiomycota*	0.0047%	0.0041%	0.0041%	0.0042%
	*Glomeromycota*	0.0020%	0.0050%	0.0034%	0.0050%
	*Blastocladiomycota*	0.0011%	0.0009%	0.0008%	0.0010%
	*Microsporidia*	0.0004%	0.0001%	0.0000%	0.0004%
	*Entomophthoromycota*	0.0004%	0.0003%	0.0002%	0.0005%
	*Cryptomycota*	0.0003%	0.0002%	0.0001%	0.0001%
Viruses	*Viruses_noname*	0.0306%	0.0250%	0.0348%	0.0286%

**Table A6:** Microbial community Shannon–Wiener indexes of different sample sites.

Sample Sites	Shannon-Wiener
BS	13.62 ± 0.04 b
MS	13.69 ± 0.08 ab
US	13.79 ± 0.05 a
OT	13.48 ± 0.08 c

Note: Different minuscule alphabet indicate divergence is significantly different at 0.05 levels.

**Table A7:** The genes related to C/N cycle and their relative abundances at different sample sites.

	BS	MS	US	OT	*p* Value	KO Description
C cycle						
K00194	4.76 × 10^−6^	4.19 × 10^−6^	5.78 × 10^−6^	3.83 × 10^−6^	0.834	acetyl-CoA decarbonylase/synthase
K00196	1.06 × 10^−6^	2.11 × 10^−6^	4.22 × 10^−6^	3.34 × 10^−6^	0.629	anaerobic carbon-monoxide dehydrogenase iron sulfur subunit
K00198	3.29 × 10^−6^	3.99 × 10^−7^	6.46 × 10^−7^	8.42 × 10^−7^	0.015	anaerobic carbon-monoxide dehydrogenase catalytic subunit
K01674	1.93 × 10^−5^	1.97 × 10^−5^	3.71 × 10^−5^	1.24 × 10^−5^	0.06	carbonic anhydrase
K03518	0.001744	0.001950	0.002013	0.001593	0.072	aerobic carbon-monoxide dehydrogenase small subunit
K03519	0.001600	0.001825	0.001950	0.001451	0.075	aerobic carbon-monoxide dehydrogenase medium subunit
K03520	0.006649	0.006648	0.006743	0.006103	0.683	aerobic carbon-monoxide dehydrogenase large subunit
K03563	3.76 × 10^−5^	3.88 × 10^−5^	3.377 × 10^−5^	4.24 × 10^−5^	0.867	carbon storage regulator
K07537	0.000102	7.09 × 10^−5^	8.59 × 10_−5_	6.33 × 10^−5^	0.103	cyclohexa-1,5-dienecarbonyl-CoA hydratase
K07539	1.35 × 10^−6^	1.43 × 10^−5^	4.12 × 10^−7^	9.83 × 10^−7^	0.482	6-oxocyclohex-1-ene-carbonyl-CoA hydrolase
K11952	0	2.80 × 10^−6^	4.52 × 10^−6^	7.81 × 10^−8^	0.165	bicarbonate transport system ATP-binding protein
K11953	5.85 × 10^−6^	2.70 × 10^−6^	9.04 × 10^−7^	4.70 × 10^−6^	0.427	bicarbonate transport system ATP-binding protein
K19066	6.86 × 10^−6^	5.36 × 10^−6^	2.53 × 10^−6^	3.66 × 10^−6^	0.686	cyclohex-1-ene-1-carbonyl-CoA dehydrogenase
K19067	1.24 × 10^−5^	1.22 × 10^−5^	8.10 × 10^−6^	2.18 × 10^−5^	0.367	cyclohexane-1-carbonyl-CoA dehydrogenase
N cycle						
K02586	1.00 × 10^−5^	2.08 × 10^−5^	8.84 × 10^−6^	8.31 × 10^−6^	0.296	nitrogenase molybdenum-iron protein alpha chain
K02588	8.15 × 10^−6^	2.00 × 10^−5^	1.26 × 10^−5^	6.73 × 10^−6^	0.161	nitrogenase iron protein NifH
K02591	4.52 × 10^−6^	1.16 × 10^−5^	6.35 × 10^−6^	2.87 × 10^−6^	0.056	nitrogenase molybdenum-iron protein beta chain
K02806	0.000237	0.000214	0.000219	0.000174	0.121	nitrogen PTS system EIIA component
K04751	0.000491	0.000530	0.000529	0.000509	0.627	nitrogen regulatory protein P-II 1
K07708	0.000334	0.000287	0.000304	0.000257	0.202	nitrogen regulation sensor histidine kinase GlnL
K07712	0.000741	0.000595	0.000674	0.000564	0.047	nitrogen regulation response regulator GlnG
K13598	0.000529	0.000470	0.000568	0.000393	0.059	nitrogen regulation sensor histidine kinase NtrY
K13599	0.0009345	0.000741	0.000909	0.000596	0.01	nitrogen regulation response regulator NtrX
K15861	9.794 × 10^−5^	5.034 × 10^−5^	3.66129 × 10^−5^	7.785 × 10^−5^	0.040	nitrogen fixation regulation protein

**Table A8:** The mantel test of different sample site.

	r	*p*
SOC	0.1884	0.1675
TN	0.6960	3 × 10^−4^
TP	−0.0546	0.5814
TK	−0.0072	0.4769
pH	0.5397	5 × 10^−4^
SWC	0.0102	0.4465
